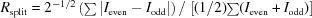# Advances in long-wavelength native phasing at X-ray free-electron lasers

**DOI:** 10.1107/S2052252520011379

**Published:** 2020-09-09

**Authors:** Karol Nass, Robert Cheng, Laura Vera, Aldo Mozzanica, Sophie Redford, Dmitry Ozerov, Shibom Basu, Daniel James, Gregor Knopp, Claudio Cirelli, Isabelle Martiel, Cecilia Casadei, Tobias Weinert, Przemyslaw Nogly, Petr Skopintsev, Ivan Usov, Filip Leonarski, Tian Geng, Mathieu Rappas, Andrew S. Doré, Robert Cooke, Shahrooz Nasrollahi Shirazi, Florian Dworkowski, May Sharpe, Natacha Olieric, Camila Bacellar, Rok Bohinc, Michel O. Steinmetz, Gebhard Schertler, Rafael Abela, Luc Patthey, Bernd Schmitt, Michael Hennig, Jörg Standfuss, Meitian Wang, Christopher J. Milne

**Affiliations:** a Photon Science Division, Paul Scherrer Institut, Forschungsstrasse 111, Villigen PSI, 5232, Switzerland; b LeadXpro AG, Park InnovAARE, Villigen, 5234, Switzerland; c Science IT, Paul Scherrer Institut, Forschungsstrasse 111, Villigen PSI, 5232, Switzerland; d Laboratory of Biomolecular Research, Division of Biology and Chemistry, Paul Scherrer Institut, Forschungsstrasse 111, Villigen PSI, 5232, Switzerland; e Institute of Molecular Biology and Biophysics, Department of Biology, ETH Zürich, Wolfgang-Pauli-Strasse 27, Zürich, 8093, Switzerland; f Department of Biology, ETH Zürich, Wolfgang-Pauli-Strasse 27, Zürich, 8093, Switzerland; g Sosei Heptares, Steinmetz Building, Granta Park, Great Abington, Cambridge CB21 6DG, United Kingdom; h Biozentrum, University of Basel, Basel, 4056, Switzerland

**Keywords:** serial femtosecond crystallography, X-ray free-electron lasers, single-wavelength anomalous diffraction, *de novo* protein structure determination, anomalous data-quality indicators

## Abstract

Long-wavelength pulses from the Swiss X-ray free-electron laser (XFEL) have been used for *de novo* protein structure determination by native single-wavelength anomalous diffraction (native-SAD) phasing of serial femtosecond crystallography (SFX) data. In this work, sensitive anomalous data-quality indicators and model proteins were used to quantify improvements in native-SAD at XFELs such as utilization of longer wavelengths, careful experimental geometry optimization, and better post-refinement and partiality correction. Compared with studies using shorter wavelengths at other XFELs and older software versions, up to one order of magnitude reduction in the required number of indexed images for native-SAD was achieved, hence lowering sample consumption and beam-time requirements significantly. Improved data quality and higher anomalous signal facilitate so-far underutilized *de novo* structure determination of challenging proteins at XFELs. Improvements presented in this work can be used in other types of SFX experiments that require accurate measurements of weak signals, for example time-resolved studies.

## Abstract 2

Significant improvements to the current state-of-the-art in phasing strategies in serial femtosecond crystallography are presented and quantified.

## 1. Introduction   

Serial femtosecond crystallography (SFX) is a unique method for macromolecular structure determination from small, weakly diffracting and radiation-sensitive crystals (Schlichting, 2015 ▸; Spence, 2017 ▸). The ultra bright and ultra short X-ray free-electron laser (XFEL) pulses allow most radiation-damage effects to be avoided in comparison with structure determination at synchrotrons (Nass, 2019 ▸). The XFEL pulses also enable time-resolved crystallography at room temperature with sub-picosecond time resolution (Barends *et al.*, 2015 ▸). Compared with synchrotron sources, ∼1 000 000 000 times higher peak brilliance of these pulses destroys the exposed microcrystal after the diffraction signal is obtained (Barty *et al.*, 2012 ▸; Lomb *et al.*, 2011 ▸). Consequently, each diffraction pattern typically originates from a different microcrystal in a random orientation. Additionally, the femtosecond duration of XFEL pulses precludes rotation of the crystal during exposure, yielding still diffraction patterns with partial Bragg reflection intensities. Retrieval of a complete data set with full reflection intensities can be achieved by assembling several thousand diffraction images (Kirian *et al.*, 2011 ▸).

The ability to obtain diffraction data from small crystals while avoiding radiation damage is particularly attractive for *de novo* phasing of difficult protein targets. However, the XFEL radiation is generated by a stochastic self-amplified spontaneous-emission process that can introduce fluctuations in the intensity, pointing direction and spectral properties of the pulses. These instabilities, combined with variations in crystal size and quality in addition to challenges in processing still diffraction patterns, introduce noise in the SFX data analysis, which complicates accurate measurement of the weak anomalous signals needed for *de novo* phasing, particularly for native single-wavelength anomalous diffraction (native-SAD) (Rose *et al.*, 2015 ▸). To decrease random errors and improve the accuracy of SFX data to a level sufficient for *de novo* phasing, an exceedingly high redundancy of measurements is mandatory (Barends *et al.*, 2014 ▸; Kirian *et al.*, 2011 ▸). While this approach has worked at XFELs, it is inefficient in terms of sample consumption and beam-time usage. Results from previous studies demonstrating *de novo* phasing using SFX data were mostly acquired from crystals diffracting strongly to high resolution (Barends *et al.*, 2014 ▸; Fukuda *et al.*, 2016 ▸; Gorel *et al.*, 2017 ▸; Hunter *et al.*, 2016 ▸; Nakane *et al.*, 2016 ▸; Yamashita *et al.*, 2017 ▸), including native-SAD phasing studies (Batyuk *et al.*, 2016 ▸; Nakane *et al.*, 2015 ▸; Nass *et al.*, 2016 ▸), which still required ≥150 000 indexed images to succeed (Table 1 ▸).

Long-wavelength X-rays can be utilized to increase the anomalous signal from naturally occurring heavy elements in proteins such as sulfur and phospho­rus (Aurelius *et al.*, 2017 ▸). This increased signal can be employed in native-SAD phasing and, in the specific case of SFX, may reduce the required number of indexed images, and thus reduce sample consumption and beam time. Additionally, the strong absorption of long-wavelength X-rays by air and large protein crystals, typically problematic at standard synchrotron macromolecular crystallography beamlines, are mitigated in SFX experiments as these are often carried out in a vacuum or helium environment with microcrystals delivered in a liquid jet or high-viscosity stream of limited diameter or on a solid support of limited thickness.

Here, we present an improvement in native-SAD phasing in SFX experiments by using longer wavelength X-rays and improved data processing. We demonstrate an up-to-tenfold reduction in the required number of indexed images for both model proteins and more challenging targets. In addition to an increased anomalous signal at longer wavelengths, we attribute this significant reduction to advances in data-analysis methods which reduced the negative impact on data quality from partial intensities and inaccurate experimental geometry in SFX data sets. This achievement can facilitate *de novo* structure determination of small and weakly diffracting protein crystals at XFELs, a potential that remains heavily underexploited.

## 2. Methods   

### 2.1. Preparation of A_2A_ receptor microcrystals   

The expression, purification and crystallization of human A_2A_ adenosine receptor (A_2A_) microcrystals were performed as previously described (Weinert *et al.*, 2017 ▸). The A_2A_ microcrystals had a plate-like shape and their average size was ∼35 × 35 × 5 µm (see Fig. S1 in the Supporting information). They were loaded into a lipidic cubic phase (LCP) injector prior to the measurements at SwissFEL directly from the Hamilton syringes in which they were grown.

### 2.2. Preparation of thaumatin microcrystals   

Thaumatin from *Thaumatococcus danielii* was purchased from Sigma–Aldrich GmbH (Buchs, Switzerland). Crystals were grown at 20°C using the vapour-diffusion method in sitting-drop CrysChem plates (Hampton Research, USA). Drops were composed of 2 µl of thaumatin solution (50 mg ml^−1^ dissolved in water) and 2 µl of reservoir solution (24% potassium sodium tartrate tetrahydrate, 100 m*M* bis-Tris propane pH 6.5) and were set up over wells containing 500 µl of reservoir solution. Crystals appeared overnight and grew over three to five days to a final size of 800 × 400 µm. The crystals were crushed in order to produce a seed stock, which was used to nucleate the growth of microcrystals as described below.

Micrometre-sized crystals were obtained by gently mixing 400 µl of thaumatin dissolved in 100 m*M* Na HEPES pH 7.0 at 88 mg ml^−1^ with 400 µl of the precipitant (1.6 *M* potassium sodium tartrate tetrahydrate, 100 m*M* Na HEPES pH 7.0) supplemented with 20 µl of the seed stock. Microcrystals grew overnight to a size of ∼20 µm in length and 10 µm in width. The microcrystalline slurry was then centrifuged at 8000*g* for two minutes and washed twice with the washing solution (0.8 *M* potassium sodium tartrate, 100 m*M* Na HEPES pH 6.8).

### 2.3. Embedding of thaumatin microcrystals in LCP   

To prepare the crystals for injection, LCP was obtained in a 100 µl Hamilton syringe by mixing 30 µl of monoolein with 20 µl of the washing solution. LCP was then mixed with 10 µl of the batch-grown microcrystals. An additional 15 µl of monoolein was added in order to keep the phase cubic.

### 2.4. Diffraction data collection   

SFX data from A_2A_ and thaumatin microcrystals were collected in August 2018 during the very first SFX experiment at SwissFEL. The high-viscosity injector designed at the Arizona State University (Weierstall *et al.*, 2014 ▸) and manufactured at the Paul Scherrer Institute (PSI) was used to deliver protein microcrystals grown or embedded in LCP to the interaction region with X-ray pulses. The injector was installed in the Prime chamber of the Alvra experimental hutch in a vertical configuration, with a distance of ∼95 mm between the nozzle tip and the detector surface. The injector was equipped with nozzles of 50 and 75 µm inner diameter for the A_2A_ and thaumatin samples, respectively. Pressure to drive the injector was supplied by a high-performance liquid chromatography (HPLC) system (Shimadzu) remotely controlled through software. The flow rate set on the HPLC pump ranged from 0.002 to 0.003 ml min^−1^ resulting in a sample consumption rate of 0.15 to 0.22 µl min^−1^. The downstream displacement of the LCP stream between two consecutive X-ray pulses was 50–75 µm and 20–30 µm (calculated) for the capillary diameters of 50 and 75 µm, respectively.

After each injector exchange and prior to the measurements the pressure in the Alvra Prime chamber was reduced to 100 mbar, then the chamber was filled with He gas to 800 mbar and reduced again to 100 mbar. The temperature in the hutch was kept constant at 24°C. An X-ray beam transmission of 2% was used for all data sets. This value was chosen to minimize disruption of the LCP column caused by interaction with X-ray pulses, to reduce the number of saturated Bragg spots and to reduce the noise originating from the background scattering while preserving high-resolution Bragg spots. Single-shot diffraction patterns were recorded using the JUNGFRAU 16M detector (Fig. S2) (Mozzanica *et al.*, 2016 ▸), with a hole in the middle for the direct beam, and saved to disk as HDF5 files containing images in the raw format as read out directly by the detector without any modifications. The data-acquisition system was operated at 25 Hz matching the repetition rate of the X-ray laser. A Kapton foil of 75 µm thickness was installed ∼20 mm from the detector surface to separate the detector compartment filled with N_2_ gas from He gas in the main experimental chamber. The pressure in these chambers was regulated to maintain equilibrium between the two sections. A detailed description of online data monitoring and offline data conversion can be found in the Supporting information.

### 2.5. Data processing, geometry optimization, phasing and refinement   

Diffraction images after applying pedestal and gain corrections were processed using *CrystFEL* version 0.7.0 (White *et al.*, 2012 ▸). Peak finding was performed on every diffraction image using the *peakfinder8* method with threshold and signal-to-noise ratio (SNR) parameters set to 50 and 5, respectively. Every diffraction image containing ten or more found Bragg peaks was identified as a ‘crystal hit’. Indexing of all data sets was performed using the *asdf* indexing algorithm method. We tested two other indexing methods available in *CrystFEL* 0.7.0: *DirAx* (Duisenberg, 1992 ▸) and *MOSFLM* (Battye *et al.*, 2011 ▸). We used the number of indexed patterns as a benchmark. We found that the number of indexed patterns was the highest for *asdf* when other parameters such as peak-finding threshold, SNR and integration radius were kept the same. We used the default values of bandwidth and divergence and the profile radius parameters automatically determined by *CrystFEL* 0.7.0 for every indexed diffraction image. The fixed geometry of the multi-segment JUNGFRAU 16M detector was refined using *geoptimizer* (Yefanov *et al.*, 2015 ▸) from the *CrystFEL* software suite. The beam centre location was optimized using peak-prediction refinement and *detector-shift* Python script from the *CrystFEL* suite.

The distance between the interaction region with X-ray pulses and the detector surface was optimized by indexing each data set separately with different detector distances and measuring the standard deviation of the distribution of the unit-cell parameters. The increment of the detector distance used in this analysis was 20 µm. The smallest standard deviation of the unit-cell parameters indicated the most optimal sample-to-detector distance as described by Nass *et al.* (2016 ▸).

Peak integration was performed using the *rings-grad* method for all data sets. Integrated reflection intensities were scaled and averaged in a Monte Carlo manner (Kirian *et al.*, 2011 ▸) using *partialator* from the *CrystFEL* software suite versions 0.8.0 and 0.7.0 with and without partiality refinement to compare the effect of post-refinement and partiality correction between different *CrystFEL* versions. The following parameters were used for merging in *partialator* with partiality and post-refinement, --model = xsphere --iterations = 1 --no-deltacchalf --push-res = 0.5; and without partiality refinement, --model = unity --iterations = 1 --no-deltacchalf --push-res = 0.5. We optimized the number of iterations (1, 2 or 3) and push-res values [infinity (the default) or 0.5] with and without the no-deltacchalf parameter, and determined the set of parameters that led to the best structural solution with the least indexed patterns for our samples and experimental conditions. The --overpredict parameter was not used. The resulting set of merged reflections was then converted to the XDS_ASCII format, passed through *XSCALE* for outlier rejection and converted to structure-factor amplitudes using *XDSCONV* from the *XDS* software package (Kabsch, 2010 ▸). The progress of the partiality refinement was monitored using the *plot-pr* and *plot-contourmap* scripts from *CrystFEL* 0.8.0 (Fig. S3).

Structure determination of the thaumatin and A_2A_ data sets was performed using the automated pipeline for substructure identification with *SHELXD* (Sheldrick, 2010 ▸), with refinement, phasing and model building implemented in the *Crank2* pipeline (Skubák & Pannu, 2013 ▸). Additionally, iterative cycles of manual model rebuilding using *Coot* (Emsley & Cowtan, 2004 ▸) and refinement using *Phenix* (Adams *et al.*, 2010 ▸) were performed to obtain final models of the A_2A_ and thaumatin molecules. The model quality was assessed using *MolProbity* (Chen *et al.*, 2010 ▸).

The peak heights from sulfur atoms in the phased anomalous difference Fourier maps of thaumatin used for the estimation of the anomalous signal strength (*S*_ano_) were calculated using the program *ANODE* (Thorn & Sheldrick, 2011 ▸), with phases from the refined model of thaumatin and reflection intensities from the whole resolution range (Table 2 ▸).

### 2.6. XFEL parameters at SwissFEL   

The pulse parameters at SwissFEL were as follows: a repetition rate of 25 Hz; a pulse-duration upper limit of 130 fs full width at half-maximum (FWHM) (estimated from a measured electron-bunch length of 130 fs FWHM); photon energies of 4.57 keV (2.713 Å) and 6.06 keV (2.046 Å) measured by monochromator scans; energy bandwidths of 0.87% (FWHM) (40 eV/4570 eV) and 0.66% (FWHM) (40 eV/6060 eV) estimated from the monochromator scans using FWHM; beam sizes of ∼8 × 8 µm at 6.06 keV and ∼10 × 10 µm at 4.57 keV focused by a pair of Kirkpatrick–Baez mirrors, measured by knife-edge scans; and pulse intensities of 5.5 × 10^15^ W cm^−2^ corresponding to 5.93 × 10^11^ photons pulse^−1^ at 4.57 keV [calculated from the 435 ± 11 µJ average pulse-energy value measured by the pulse-intensity monitor (Juranić *et al.*, 2018 ▸)] and 8 × 10^15^ W cm^−2^ corresponding to 4.15 × 10^11^ photons pulse^−1^ at 6.06 keV (402 ± 43 µJ). A beamline transmission of ∼50% and an attenuation factor of 98%, used deliberately to avoid jet instabilities and to reduce background scattering as described earlier, were not included in the above calculations.

### 2.7. Data and materials availability   

We deposited all diffraction data that contained crystal hits reported in this study in the Coherent X-ray Imaging Data Bank (CXIDB) with the accession codes ID 103, ID 104 and ID 105 (Maia, 2012 ▸). The atomic coordinates and the structure-factor amplitudes have also been deposited in the Protein Data Bank (PDB) under the accession codes highlighted in this article.

## 3. Results   

### 3.1. Experimental setup and data-collection parameters   

SFX data sets from microcrystals of thaumatin and human A_2A_ adenosine receptor (A_2A_) were measured using X-ray wavelengths of 2.046 Å (6.06 keV) and 2.713 Å (4.57 keV) at the SwissFEL (Milne *et al.*, 2017 ▸), and recorded with the new JUNGFRAU 2D X-ray detector that allows the acquisition of excellent quality data (Mozzanica *et al.*, 2016 ▸). The microcrystals were grown (A_2A_) or embedded (thaumatin) in LCP and injected into the interaction region with focused X-ray pulses in the Alvra Prime experimental chamber at SwissFEL using an LCP injector (see *Methods* [Sec sec2]). We collected 271 609 indexable diffraction images from thaumatin crystals at 6.06 keV and 242 578 images at 4.57 keV, and 199 136 images from A_2A_ crystals at 4.57 keV at a repetition rate of 25 Hz (Table 2 ▸). We evaluated the data quality while systematically decreasing the number of images and determined the minimum for native-SAD phasing and automatic model building (see *Methods* [Sec sec2] and the Supporting information).

### 3.2. *De novo* structure determination of thaumatin at 6.06 keV   

We first performed native-SAD phasing of thaumatin data collected at 6.06 keV with all the images. To find the minimum number of images for successful and fully automatic structure determination, we systematically decreased the size of our data set, leading to 50 000 patterns required. The overall and split into resolution bins data-quality indicators from *CrystFEL* of the whole and minimal thaumatin 6.06 keV data sets are given in Tables S1–S3 in the Supporting information. The sub-structure determination, phasing and automatic model building were accomplished using the *Crank2* pipeline and resulted in 97% of the model being built automatically (see *Methods* [Sec sec2] and Figs. S4–S7). The resolution of our data set extended to 1.95 Å; however, data to 2.8 Å were sufficient for *de novo* structure determination.

### 3.3. *De novo* structure determination of thaumatin at 4.57 keV   

In an identical setup, we carried out a native-SAD experiment using thaumatin microcrystals at 4.57 keV to exploit the enhanced imaginary part of the anomalous scattering factor of sulfur atoms (*f*′′ = 1.51 e^−^ at 4.57 keV versus *f*′′ = 0.95 e^−^ at 6.06 keV). Here, 20 000 indexed diffraction patterns were already sufficient for *de novo* structure determination at 2.65 Å resolution and model building using *Crank2*, with 92% of the model built automatically (see *Methods* [Sec sec2], Tables S4–S6 and Figs. S8–S11). The maximal attainable resolution of this data set was limited to 2.65 Å because of geometrical constraints of the experimental setup and wavelength.

### 3.4. Comparison of anomalous signal strength from thaumatin at 6.06 and 4.57 keV   

**Figure 1:**
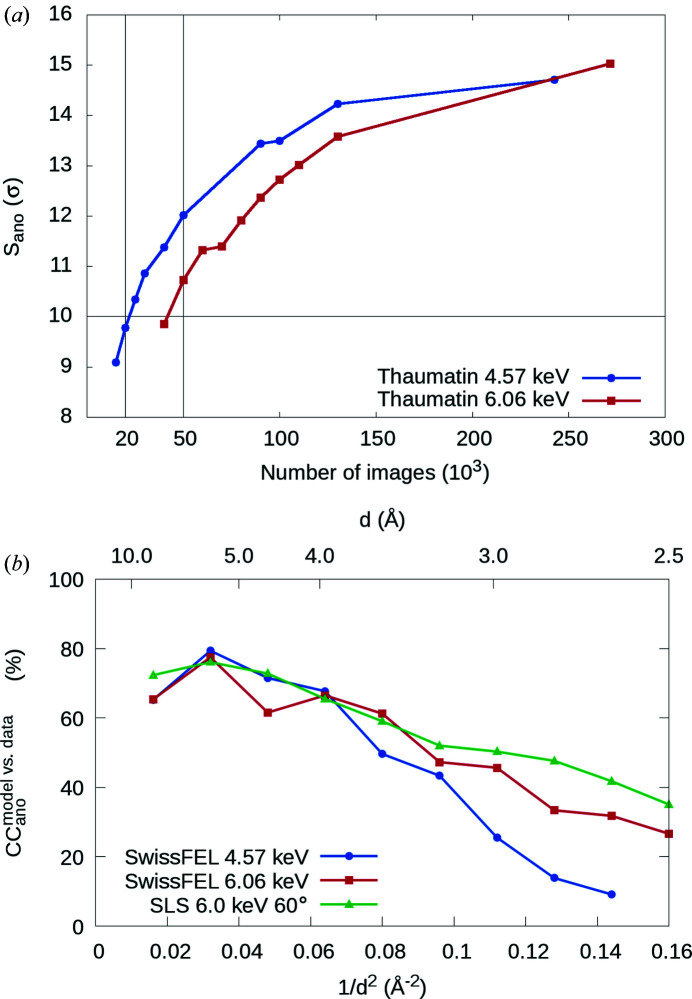
Anomalous data-quality indicators. (*a*) Anomalous signal strength (*S*_ano_) of thaumatin 4.57 and 6.06 keV data sets for different numbers of indexed diffraction images. The horizontal line indicates an *S*_ano_ value of 10, below which SAD phasing is difficult. The two vertical lines indicate the minimal numbers of indexed images required for structure determination using native-SAD in this study. (*b*) Correlation coefficient between the measured and calculated anomalous difference structure-factor amplitudes (CC_ano_^model versus data^) for thaumatin data sets with minimal amount of data necessary for successful structure solution using native-SAD at the SwissFEL and the Swiss Light Source (SLS) (Leonarski *et al.*, 2018 ▸).

To quantify the effect of lower photon energy on the anomalous signal in SFX, we estimated the magnitude of the anomalous signal contained in our thaumatin data sets. We calculated the average peak heights of the phased anomalous Fourier difference maps (*S*_ano_) for different numbers of indexed diffraction images at two photon energies, 6.06 and 4.57 keV [Fig. 1 ▸(*a*)]. *S*_ano_ is an unbiased data-quality indicator that allows direct comparison of the quality and accuracy of the measured data sets that contain anomalous signal. An *S*_ano_ value of 10 or higher typically indicates whether the structure can be determined using SAD phasing (Terwilliger *et al.*, 2016 ▸). In accordance with the expected 2.5-fold enhancement of *f*′′ [(1.51/0.95)^2^ = 2.5] at lower photon energy, the anomalous signal strength (*S*_ano_) from sulfur atoms in thaumatin at 4.57 keV is higher than at 6.06 keV for data sets with the same number of indexed images. Consequently, 2.5 times fewer diffraction images were required to determine the structure using native-SAD phasing at 4.57 keV. At both photon energies, the thaumatin data sets with the minimum number of indexed images required to solve the structure had *S*_ano_ values of ∼10 [Fig. 1 ▸(*a*), horizontal and vertical lines].

### 3.5. Comparison of anomalous data-quality indicators for thaumatin data sets with minimal number of indexed images required to determine the structure   

To further investigate the anomalous data-quality indicators, we calculated the correlation coefficients between the observed (|Δ*F*_obs_|) and the expected (|ΔF_calc_|) anomalous difference structure-factor amplitudes (CC_ano_^model versus data^) from data sets with the minimal number of images. We compared it with the CC_ano_^model versus data^ obtained from a synchrotron-rotation data set from a thaumatin crystal collected at 6 keV photon energy with a JUNGFRAU detector (Leonarski *et al.*, 2018 ▸) [Fig. 1 ▸(*b*)]. The CC_ano_^model versus data^ values were calculated using a custom script that is available upon request. The synchrotron data set contains the minimum amount of data necessary for successful native-SAD phasing (60°) (Leonarski *et al.*, 2018 ▸). Indeed, the 6.06 keV SwissFEL data set with 50 000 patterns has a similar CC_ano_^model versus data^ to the synchrotron data, indicating that the anomalous signal has been accurately extracted from our SFX data set. The 4.57 keV SFX data has comparable anomalous signal strength to 3.5 Å resolution, from which point on it gradually deteriorates toward higher resolution. The decrease of the CC_ano_^model versus data^ of the 4.57 keV thaumatin SFX data set at resolutions higher than 3.5 Å was primarily caused by excessive absorption of X-rays (up to 50%) at high diffraction angles by the 75 µm Kapton window mounted in front of the JUNGFRAU 16M detector. The Kapton window was used to separate the detector from the He atmosphere in the chamber. Nevertheless, high-resolution data were not required for sub-structure determination and data up to 2.8 Å were sufficient for density modification and automatic model building in *Crank2*.

### 3.6. Optimization of the sample-to-detector distance   

The indexing success rate and accuracy largely depend on the precise knowledge of the experimental geometry. Systematic errors introduced during data collection, such as slightly different detector distances between sample changes, can negatively affect the final data quality (Nass *et al.*, 2016 ▸). Reduction of such errors is especially important in cases where high accuracy in the measurement is necessary, *e.g.* in *de novo* phasing and time-resolved SFX experiments. In order to reduce the negative influence of systematic errors in our data sets, we optimized the positions of the individual detector modules, determined the optimal beam-centre position using peak-prediction refinement protocol implemented in *CrystFEL* and optimized the sample-to-detector distance (see *Methods* [Sec sec2]).

**Figure 2:**
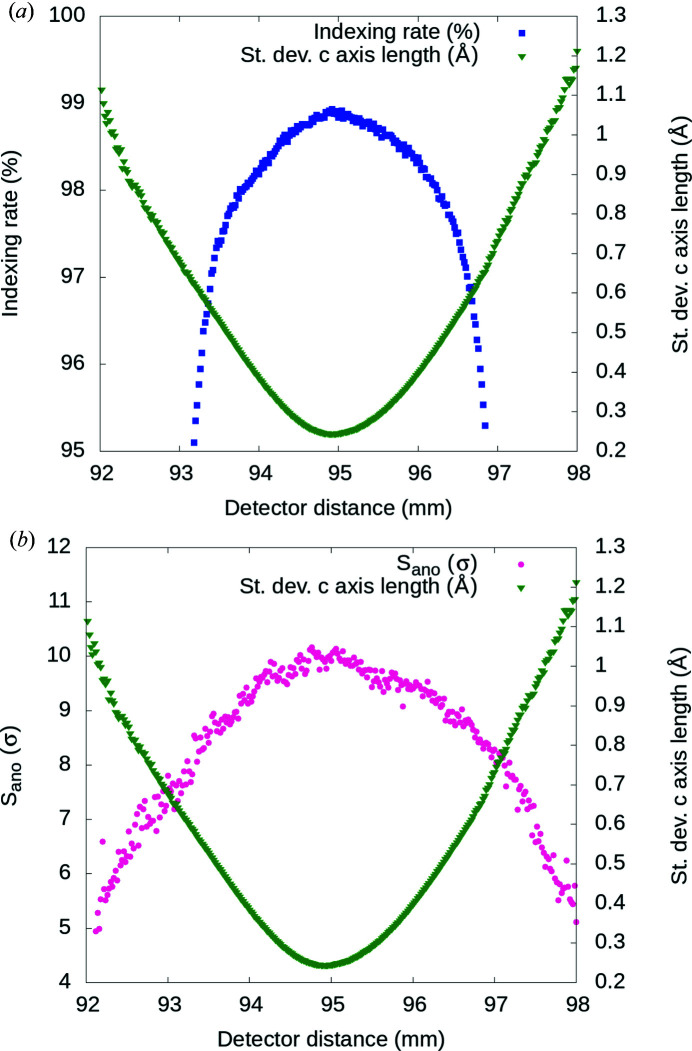
Detector-distance optimization using thaumatin crystals. (*a*) The smallest standard deviation of the distribution of the unit-cell parameters (green triangles) indicates the optimal sample-to-detector distance 94.9 mm. The indexing rate (blue squares) is highest at the optimal detector distance. (*b*) Average peak height of the anomalous Fourier difference map (*S*_ano_) from thaumatin at 4.57 keV (pink circles) calculated at different detector distances. *S*_ano_ is highest at the optimal detector distance which corresponds to the smallest standard deviation of *c* axis length (green triangles).

In order to find the optimal detector distance, we calculated the standard deviation of the distribution of the unit-cell parameters for each sample change at different detector distances. Fig. 2 ▸(*a*) shows the standard deviation of unit-cell length *c* and the indexing rate for different detector distances for one of the thaumatin 4.57 keV samples. This batch contained 25 821 selected diffraction images with crystal hits, up to 98.9% of which could be indexed at the optimal detector distance. The optimal detector distance, which corresponds to the minimum of the standard deviation of the distribution of the unit-cell length *c*, was 94.9 mm. Analysis of other unit-cell parameters yielded the same results. The optimal detector distance also corresponded to the highest indexing rate. We observed that the quality of the indexing solution directly relates to the accuracy of the measured structure-factor amplitudes, and hence to the strength of the anomalous signal (*S*_ano_), which in this sample batch was highest for the optimized detector distance of 94.9 mm [Fig. 2 ▸(*b*)]. Data sets for all sample batches indexed using optimized sample-to-detector distances were merged together to create final data sets with all indexed images.

### 3.7. Comparison of post-refinement and partiality correction procedures in different *CrystFEL* versions   

Partially recorded Bragg spot intensities are intrinsic to every SFX data set. In most SFX experiments performed to date, full reflection intensities were determined by averaging a large enough number of partial measurements of the same reflection from different crystals (Kirian *et al.*, 2010 ▸, 2011 ▸). An estimation of the partiality of each such reflection in the presence of random and systematic errors has been notoriously difficult, although various partiality refinement protocols have been developed (Ginn *et al.*, 2015 ▸; Kabsch, 2014 ▸; Sauter, 2015 ▸; White, 2014 ▸). One of the pre-requisites for accurate partiality estimation is precise knowledge of the experimental geometry (White, 2014 ▸). After optimizing the geometrical parameters of our experimental setup as described above, we investigated whether we could improve data quality with the recent developments in the *CrystFEL* post-refinement procedures, such as advanced scaling and partiality correction (White *et al.*, 2016 ▸).

**Figure 3:**
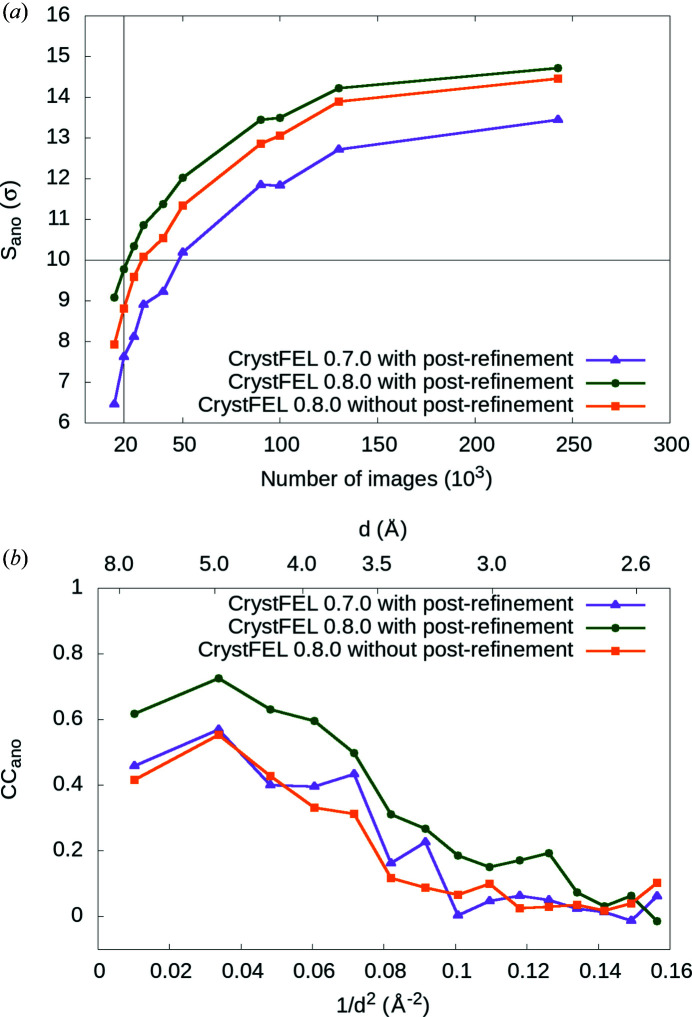
Comparison of the anomalous signal strength (*S*_ano_) and anomalous correlation coefficient (CC_ano_) for thaumatin at 4.57 keV. (*a*) Data sets with different numbers of images were processed using old and new versions of *partialator * from the *CrystFEL* software suite, with and without post-refinement and partiality correction. *S*_ano_ is significantly larger when the newer version of post-refinement and partiality correction is used (green circles), as compared with the older version (purple triangles) and the newer version with only scaling (orange squares). (*b*) Similarly, CC_ano_ from 50 000 randomly selected thaumatin 4.57 keV diffraction images is higher when the newer version of *CrystFEL* with post-refinement and partiality correction is used on the same data set (green circles).

We used data-quality indicators sensitive to the overall accuracy of the data set, anomalous signal strength (*S*_ano_) and anomalous correlation coefficient (CC_ano_) to quantify whether the improvements in the post-refinement protocols implemented in *CrystFEL* version 0.8.0 with respect to the previous *CrystFEL* version 0.7.0 have an influence on the accuracy of the structure factors in our final data sets. We calculated *S*_ano_ values for thaumatin 4.57 keV data sets with different numbers of indexed images using *CrystFEL* versions 0.7.0 and 0.8.0 with scaling, post-refinement and partiality correction and using *CrystFEL* 0.8.0 without post-refinement and partiality correction but with scaling [Fig. 3 ▸(*a*)]. We observed that *S*_ano_ values were higher for the same number of images when the newer *CrystFEL* version was used to merge partial reflections into the final data set. Within the newer *CrystFEL* version, we also observed that the post-refinement with partiality correction further improved the quality of the data sets as indicated by the highest *S*_ano_ values (Table S7).

To quantify whether CC_ano_ also improved when using the newer *CrystFEL* version, we used 50 000 randomly selected indexed diffraction images from the thaumatin 4.57 keV data set and calculated CC_ano_ values as a function of the resolution [Fig. 3 ▸(*b*)]. Here, we observed that *CrystFEL* 0.8.0 with post-refinement and partiality correction resulted in higher CC_ano_ values indicating that the improvements implemented in *CrystFEL* 0.8.0 have a positive effect on the accuracy of the data set.

### 3.8. *De novo* structure determination of A_2A_ at 4.57 keV   

**Figure 4:**
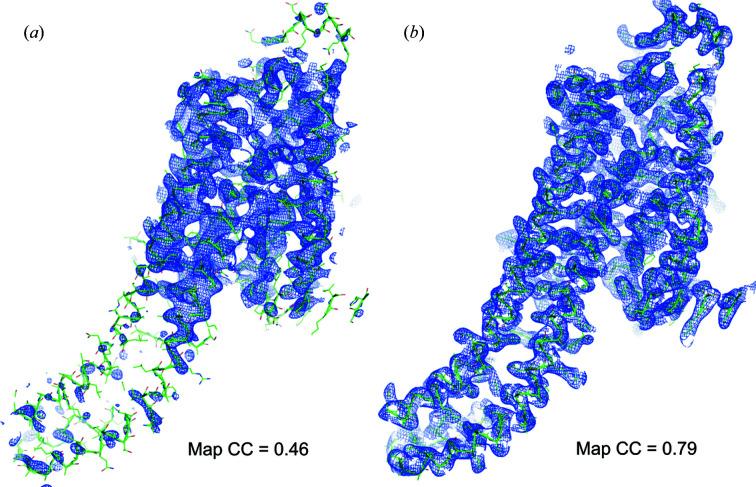
Phasing and automatic model building. (*a*) The experimental electron-density map (contoured at 1.0σ) after phasing and density modification obtained from the A_2A_ data set with a minimal number of indexed images (50 000) at 4.57 keV superposed with the A_2A_ molecule after automatic model building and refinement. (*b*) The 2*mF*_o_ − *DF*_c_ electron-density map after automatic model building and refinement. The MapCC values indicate the correlation coefficient between the map shown in the figure and the final refined map.

After establishing the improvements in native-SAD phasing with the thaumatin SFX data sets, we applied native-SAD at 4.57 keV to a membrane-protein target, the human A_2A_ adenosine receptor, as a control with qualities closer to a more realistic sample. Although the A_2A_ crystals diffracted strongly beyond the geometrically imposed resolution limit (2.65 Å), they have a higher overall *B* factor than thaumatin crystals, therefore representing a more difficult test case. Using all available indexed diffraction patterns (199 136), the structure of A_2A_ was determined readily. With only 50 000 indexed images of A_2A_ processed with individual crystal-dependent resolution cut-offs to suppress noise at high resolution from weak data, the positions of sulfur atoms were identified by *SHELXD*, and the iterative density modification and model building in *Crank2* built an almost complete model (403 of 433 residues) (see Fig. 4 ▸, *Methods* [Sec sec2], Tables S8–S11 and Figs. S12–S15).

## 4. Discussion   

Here, we have applied long-wavelength XFEL pulses from SwissFEL and advances in data analysis to determine the structures of thaumatin and the A_2A_ adenosine receptor using native-SAD phasing. Moreover, we provide a comprehensive analysis of factors that improved these native-SAD results. While a direct comparison of SAD phasing results obtained at different facilities using different detectors, machine properties and experimental setups may be constrained by the many differences, a careful comparison can still be informative.

The improvements presented in this work are primarily caused by a combination of the following factors: (i) an increased anomalous signal from sulfur atoms at a longer X-ray wavelength, (ii) a careful optimization of the experimental geometry that decreased systematic errors, (iii) an improved post-refinement and partiality correction in the *CrystFEL* 0.8.0 software suite and (iv) an accurate measurement of the diffraction signals using the low-noise high-dynamic range JUNGFRAU 16M detector (Mozzanica *et al.*, 2016 ▸). We addressed the first three contributing factors using thaumatin data sets.

First, we compared the effect of a longer wavelength on the anomalous signal strength (*S*_ano_) and the effectiveness of automatic structure determination of thaumatin for different numbers of patterns collected at 4.57 and 6.06 keV at SwissFEL (Fig. 1 ▸). We needed only 20 000 diffraction images at 4.57 keV compared with 50 000 at 6.06 keV to determine the structure using fully the automatic native-SAD pipeline from *Crank2*, despite the fact that the resolution of the 4.57 keV data set was geometrically limited to 2.65 Å. This result suggests that using a longer XFEL wavelength for native-SAD phasing results in lower sample consumption and beam-time usage, by a factor of 2.5 in our case.

Next, we investigated whether systematic errors such as incorrect detector distance and geometry have a negative impact on the anomalous signal strength and the quality of indexing. We found that *S*_ano_ and the indexing rate is maximized when the spread of the unit-cell parameters is smallest, indicating that the detector distance should be optimized for each sample batch to reduce systematic errors and to improve the accuracy of the final data set (Fig. 2 ▸). A similar approach to improve the integrated structure-factor intensity and heavy-atom anomalous peak heights from XFEL data by refining the experimental-geometry parameters was successfully applied in other studies (Brewster *et al.*, 2018 ▸; Nass *et al.*, 2016 ▸). Other sources of random error that negatively influence the precision of SFX data are shot-to-shot fluctuations of the X-ray pulses (*e.g.* pulse intensity, wavelength spread, spiky spectra, pulse duration) and crystal-to-crystal differences (*e.g.* size distribution, non-isomorphism). Most of the SFX studies to date have relied on averaging enough measurements of the same reflection in a Monte Carlo manner to minimize these errors. The same approach was applied to overcome the difficulty of retrieving full Bragg spot intensities from partially recorded reflections in still diffraction patterns from crystals in random orientations. Thirdly, we identified that the upgraded post-refinement and partiality corrections introduced in the *CrystFEL* version 0.8.0 software suite improved the accuracy of the final structure-factor intensities when compared with the previous version. In particular, the *S*_ano_ and CC_ano_ values were higher in thaumatin data sets when the newer software version was used, which allowed us to determine the structures *de novo* using fewer images (Fig. 3 ▸).

Finally, we applied the improved methods in native-SAD phasing at XFELs identified at SwissFEL using the model system thaumatin to a more challenging target, the A_2A_ human adenosine receptor. This system represents a class of membrane proteins, G-protein-coupled receptors (GPCRs), which, in general, pose a challenge for structure determination because they are characterized by a higher flexibility (temperature factor) and thus higher crystal disorder. In this case, we used up to one order of magnitude fewer images of the A_2A_ adenosine receptor for native-SAD phasing compared with what was needed previously at an XFEL (Batyuk *et al.*, 2016 ▸). Importantly, in both studies the resolution of the data sets was similar because of geometrical limitations imposed by the experimental setup and wavelength, which excludes the impact of different high-resolution limits on the performance of structure determination (Yamashita *et al.*, 2017 ▸). This significant improvement opens up an opportunity for native-SAD phasing of small and radiation-sensitive membrane protein crystals with significantly reduced sample consumption and beam time.

In the case of thaumatin, we achieved a 2.5-fold improvement of the number of patterns required for structure determination at 4.57 keV compared with 6.06 keV using the same experimental setup at SwissFEL, and a threefold improvement when compared with a thaumatin native-SAD phasing study at 6 keV, compared with previous XFEL measurements (Nass *et al.*, 2016 ▸). This is also the lowest number of indexed diffraction patterns needed for native-SAD in SFX to date, which is approaching those required for *de novo* phasing using seleno­methio­nine incorporation or heavy-metal derivatization in previously reported SFX studies (Table 1 ▸).

We attribute this remarkable achievement to the collective technological improvements enabling the use of longer-wavelength XFEL pulses for SFX and better data-analysis methods. Native-SAD phasing using long-wavelength XFEL pulses provides a compelling strategy to obtain unbiased experimental phases when structure determination is difficult because of inaccurate molecular-replacement models, particularly with low-resolution data. This work suggests that long-wavelength native-SAD experiments at XFELs have the potential to extend the scope of attainable novel structures in cases where radiation damage or poor phase quality is limiting the structure determination at synchrotrons. This is particularly important for membrane proteins that do not crystallize readily to a size sufficient for synchrotron studies. Several other XFEL facilities worldwide are capable of reaching X-ray beam parameters including the tender X-ray energy regime (2–5 keV), providing excellent opportunities for long-wavelength native-SAD phasing of difficult targets. Recent developments, such as the multivariate Bayesian model for estimating anomalous differences (Garcia-Bonete & Katona, 2019 ▸) and improvements to the error estimates of XFEL data (Brewster *et al.*, 2019 ▸), could potentially improve the SAD phasing of XFEL data further. Moreover, the improved data-analysis methods implemented in *CrystFEL* version 0.8.0 and detector-distance optimization protocols can be applied to all types of SFX studies, not only to SAD phasing.

## Supplementary Material

Supporting information. DOI: 10.1107/S2052252520011379/zf5013sup1.pdf

PDB reference: A_2A_, 4.57 keV, all diffraction images, 6s0l

PDB reference: A_2A_, 4.57 keV, 50 000 diffraction images, 6s0q

PDB reference: thaumatin, 4.57 keV, all diffraction images, 6s19

PDB reference: thaumatin, 4.57 keV, 20 000 diffraction images, 6s1d

PDB reference: thaumatin, 6.06 keV, all diffraction images, 6s1e

PDB reference: thaumatin, 6.06 keV, 50 000 diffraction images, 6s1g

## Figures and Tables

**Table 1:** A summary of *de novo* phasing SFX studies and selected *de novo* phasing serial crystallography studies at synchrotron sources

Sample	Element	Bijvoet ratio (%)	Phasing method	Minimal number of images	Resolution (Å)	Photon energy (keV)	Facility	Publication
A_2A_	S	2.5	SAD	50000	2.65	4.57	SwissFEL	This work
A_2A_	S	1.5	SAD	500000	2.5	6.0	LCLS	Batyuk *et al.* (2016 ▸)
A_2A_	S	1.5	SAD	243537	2.67	6.0	SLS	Weinert *et al.* (2017 ▸)
Thaumatin	S	3.2	SAD	20000	2.65	4.57	SwissFEL	This work
Thaumatin	S	2.0	SAD	50000	1.95	6.06	SwissFEL	This work
Thaumatin	S	2.0	SAD	150000	2.1	6.0	LCLS	Nass *et al.* (2016 ▸)
Lysozyme	S + Cl	1.9	SAD	150000	2.1	7.0	SACLA	Nakane *et al.* (2015 ▸)
Se-B SA	Se	1.2	SAD	481079	1.9	12.8	LCLS	Hunter *et al.* (2016 ▸)
Stem	Se	3.7	SAD	13000	1.4	13.0	SACLA	Yamashita *et al.* (2017 ▸)
ACG	Se	2.2	SAD	60000	1.5	13.0	SACLA	Yamashita *et al.* (2017 ▸)
CuNiR	Cu	1.6	SAD	155588	1.6	10.8	SACLA	Fukuda *et al.* (2016 ▸)
LRE	Hg	4.2	SAD	11000	1.5	12.6	SACLA	Yamashita *et al.* (2017 ▸)
LRE	Hg	4.2	SIRAS	18000	1.7	12.6	SACLA	Yamashita *et al.* (2015 ▸)
BinAB	Hg, Gd, I	–	MIRAS	357765	2.4, 2.35, 2.6	8.8	LCLS	Colletier *et al.* (2016 ▸)
bR	I	10.5	SAD	23347	2.1	7.0	SALCA	Nakane *et al.* (2016 ▸)
Lysozyme	Gd	10.2	SAD	5000	1.9	9.0	SACLA	Gorel *et al.* (2017 ▸)
Lysozyme	Gd	10.2	MAD	5000	1.9, 2.0	9.0 + 7.0	SACLA	Gorel *et al.* (2017 ▸)
Lysozyme	Gd	11.0	SAD	60000	2.1	8.5	LCLS	Barends *et al.* (2014 ▸)
Proteinase K	Pr	4.8	SAD	3000	1.5	10	SACLA	Sugahara *et al.* (2017 ▸)
Proteinase K	Pr	4.8	SIRAS	2000	1.5	10	SACLA	Sugahara *et al.* (2017 ▸)

**Table 2:** SFX data-collection and refinement statistics

Data set	A_2A_		Thaumatin		Thaumatin	
	All	50000	All	50000	All	20000
Wavelength, photon energy (Å, keV)	2.713, 4.57		2.046, 6.06		2.713, 4.57	
Temperature (K)	297		297		297	
Space group	*C*222_1_		*P*4_1_2_1_2		*P*4_1_2_1_2	
Unit-cell dimensions *a, b, c* (Å)	40.34 ± 0.08, 180.66 ± 0.37, 143.05 ± 0.33		58.32 ± 0.13, 58.32 ± 0.11, 150.90 ± 0.24		58.50 ± 0.14, 58.50 ± 0.14, 151.25 ± 0.24	
No. of collected patterns	2251787	—	1169996	—	1476000	—
No. of indexed patterns (% of collected)	199136 (8.8)	50000	271609 (23.2)	50000	242578 (16.4)	20000
No. of unique reflections	29351	29281	36568	33828	15488	15488
Resolution (Å)†	35.71–2.65 (2.71–2.65)	35.71–2.65 (2.71–2.65)	24.39–1.95 (2.00–1.95)	24.39–2.00 (2.05–2.00)	35.71–2.65 (2.71–2.65)	35.71–2.65 (2.71–2.65)
Completeness (%)	100 (100)	100 (100)	100 (100)	100 (100)	100 (100)	100 (100)
Average redundancy	858 (293)	193 (13)	1149 (280)	188 (13)	1292 (384)	125 (37)
CC_1/2_	0.999 (0.707)	0.997 (0.453)	0.999 (0.732)	0.998 (0.451)	0.999 (0.864)	0.995 (0.856)
CC†	0.999 (0.910)	0.999 (0.790)	0.999 (0.919)	0.999 (0.788)	0.999 (0.963)	0.999 (0.961)
CC_ano_	0.568 (0.086)	0.318 (0.073)	0.640 (0.279)	0.296 (−0.009)	0.877 (0.207)	0.387 (0.049)
Overall 〈*I*/σ(*I*)〉	23.29 (0.70)	12.70 (0.97)	23.96 (0.65)	11.81 (1.17)	34.97 (0.99)	11.14 (0.79)
*R*_split_ (%)‡	2.26 (135.73)	4.61 (109.68)	1.97 (134.86)	4.81 (95.33)	1.72 (75.81)	5.93 (111.57)
Refinement						
Resolution (Å)	34.50–2.65	34.50–2.65	23.15–1.95	23.15–2.00	31.98–2.65	31.98–2..65
Number of reflections used in refinement	29238	29312	36026	33556	14496	14552
*R*_work_/*R*_free_	0.1974/0.2203	0.1939/0.2210	0.1441/0.1681	0.1393/0.1692	0.1503/0.1870	0.1529/0.1962
Number of atoms	3286	3286	1622	1656	1563	1563
Protein	2998	2998	1558	1558	1553	1553
Ligand	288	288	10	10	10	10
Water	0	0	54	79	0	0
Overall *B* factor (Å^2^)	117.6	96.6	56.0	44.6	85.9	86.2
R.m.s. deviations						
Bond lengths (Å)	0.011	0.010	0.007	0.008	0.008	0.008
Bond angles (°)	1.664	1.593	0.817	0.978	0.932	0.931
Ramachandran plot (%)						
Most favoured	97.66	97.66	98.54	97.56	96.59	96.59
Additionally allowed	2.34	2.34	1.46	2.44	3.41	3.41
Disallowed	0	0	0	0	0	0

† Numbers in parentheses refer to the highest resolution shell.

‡