# Supplementary material for: Advances in long-wavelength native phasing at X-ray free-electron lasers
Source: IUCrJ. 2020 Sep 9;7(Pt 6):965–75. doi: 10.1107/S2052252520011379 (PMC7642782; doi:10.1107/S2052252520011379)
Supplement: Supplementary file 1 [file m-07-00965-sup1.pdf]

IUCrJ

Volume 7 (2020)

Supporting information for article:

Advances in long-wavelength native phasing at X-ray free-electron lasers

Karol Nass, Robert Cheng, Laura Vera, Aldo Mozzanica, Sophie Redford, Dmitry Ozerov, Shibom Basu, Daniel James, Gregor Knopp, Claudio Cirelli, Isabelle Martiel, Cecilia Casadei, Tobias Weinert, Przemyslaw Nogly, Petr Skopintsev, Ivan Usov, Filip Leonarski, Tian Geng, Mathieu Rappas, Andrew S. Doré, Robert Cooke, Shahrooz Nasrollahi Shirazi, Florian Dworkowski, May Sharpe, Natacha Olieric, Camila Bacellar, Rok Bohinc, Michel O. Steinmetz, Gebhard Schertler, Rafael Abela, Luc Patthey, Bernd Schmitt, Michael Hennig, Jörg Standfuss, Meitian Wang and Christopher J. Milne

S1. Online data monitoring and offline data conversion

Data from 31 modules of the Jungfrau 16M detector (Mozzanica *et al.*, 2016) were read out by a custom detector backend software at a rate of 775 MB/s. After assembling the frame from the individual modules, the images were streamed using the *zeromq* protocol to the data writer (25 Hz, all images), to the online visualization (1 Hz, one out of 25 images) and online analysis processes (5 Hz, every 5th image). The writer saved the images in the uncorrected, raw format (directly as it is read out by the detector) in hdf5 files, one file per run. Unless interrupted, most of the runs had 10,000 images. For online visualization the *bokeh* library [http://bokeh.pydata.org/] was used to display an assembled image in a web-browser after detector corrections (pedestal subtraction, gain correction, bad pixel identification and masking). The online visualization functions included: maintaining a buffer with 60 images, zoom and pan functions to inspect any area of interest in the image and reporting about time-based and frame-based image characteristics, such as the integrated intensity of the whole image, pixel or area of interest. In the online analysis processes, each received image was analyzed using a custom version of the *peakfinder8* algorithm from the CrystFEL software suite (White *et al.*, 2012) for the presence of Bragg spots. Images with equal to or more than 15 identified spots were regarded as crystal hits. Information about the position of spots in an image and the number of crystal hits were sent to another online visualization process, which displayed information on the percentage of hits and the diffraction resolution limit. The peak detection parameters of the *peakfinder8* algorithm such as the number of connected pixels, intensity threshold and signal to noise ratio needed to be adjusted for each sample and data collection settings. The online hit detection was used to identify and correct misalignments of the jet with respect to the beam position and to estimate the amount of collected data from a given sample. After the image was recorded by the Jungfrau 16M detector, it took approximately 1 second for this image to be displayed in the online visualization. For the offline data analysis, the raw diffraction images were converted to the corrected, unassembled format by applying gain and pedestal corrections and thereafter the images were saved as hdf5 files. The pedestal values were calculated from dark runs, taken approximately every 24 hours during the experiment. The conversion factors (gain factors) for each pixel and gain setting used to transform ADU values to keV were obtained from the SLS Detector group that manufactured the JUNGFRAU 16M detector.

S2. Structure determination of thaumatin from data set acquired at 6.06 keV by native-SAD

The structure of thaumatin was determined to 1.95 Å resolution from all available indexed images (271,609) collected at 6.06 keV by the automated pipeline for structure determination implemented in *CRANK2* pipeline (Skubak & Pannu, 2013). The heavy atom substructure was found by *SHELXD* (Sheldrick, 2010) after 616 trials searching for 9 sites with resolution cut-off of 3.5 Å. The CFOM of the best solution was 68.3, CC_{all} was 44.5 and CC_{weak} was 23.8 (Supplementary Figure 3). The combined model building and refinement finished after 212 residues were built in 3 fragments, 97.1 %

of them were assigned to the sequence (Supplementary Figure 4). The refinement R_{work} and R_{free} factors of the automatically built thaumatin model were 24.9 % and 29.6 % respectively. The average peak height of the phased anomalous Fourier difference map for the first 17 highest peaks obtained with the fully refined model was 14.6.

The structure of thaumatin from the minimal number of indexed images (50,000) was determined to 2.0 Å resolution using the *CRANK2* pipeline. The heavy atom substructure was found by *SHELXD* after 4171 trials searching for 10 sites with resolution cut-off of 3.5 Å and additional parameters ESEL = 1.3 and MIND = (-3.5, 2.8). Lower than default ESEL values usually work better for low-resolution data sets (http://shelx.uni-ac.gwdg.de/~athorn/pdf/thorn2017a.pdf). The MIND parameter defines the minimal distance (in Å) between heavy atoms (HA) sites. The above setting of MIND parameter ensured that disulphides were treated as single HA. The CFOM of the best solution was 56.8, CC_{all} was 39.3 and CC_{weak} was 17.5 (Supplementary Figure 5). The combined model building and refinement finished after 202 residues were built in 1 fragment, 97.1 % of them were assigned to the sequence (Supplementary Figure 6). The refinement R_{work} and R_{free} factors of the automatically built thaumatin model were 27.6 % and 32.7 % respectively. The average peak height of the phased anomalous Fourier difference map for the first 17 highest peaks obtained with the fully refined model was 9.57.

S3. Structure determination of thaumatin from data set acquired at 4.57 keV by native-SAD

The structure of thaumatin was determined to 2.65 Å resolution from all available indexed images (242,578) collected at 4.57 keV by the automated pipeline for structure determination implemented in *CRANK2* pipeline. The heavy atom substructure was found by *SHELXD* after 78 trials searching for 9 sites with resolution cut-off of 3.5 Å. The CFOM of the best solution was 77.4, CC_{all} was 49.3 and CC_{weak} was 28.1 (Supplementary Figure 7). The combined model building and refinement finished after 211 residues were built in 6 fragments, 92.7 % of them were assigned to the sequence (Supplementary Figure 8). The refinement R_{work} and R_{free} factors of the automatically built thaumatin model were 28.1 % and 35.2 % respectively. The average peak height of the phased anomalous Fourier difference map for the first 9 highest peaks obtained with the fully refined model was 14.5.

The structure of thaumatin from the minimal number of indexed images (20,000) was determined to 2.65 Å resolution using the *CRANK2* pipeline. The heavy atom substructure was found by *SHELXD* after 9738 trials searching for 9 sites with resolution cut-off of 3.0. The CFOM of the best solution was 41.6, CC_{all} was 27.2 and CC_{weak} was 14.4 (Supplementary Figure 9). The combined model building and refinement finished after 207 residues were built in 5 fragments, 90.3 % of them were assigned to the sequence (Supplementary Figure 10). The refinement R_{work} and R_{free} factors of the automatically built thaumatin model were 31.3 % and 40.7 % respectively. The average peak height

of the phased anomalous Fourier difference map for the first 9 highest peaks obtained with the fully refined model was 9.82.

S4. Structure determination of A_{2A} from data set acquired at 4.57 keV by native-SAD

The structure of A_{2A} was determined to 2.65 Å resolution from all available indexed images (199,136) collected at 4.57 keV using the automated pipeline for structure determination implemented in *CRANK2* (Online Methods). The heavy atom substructure was found by *SHELXD* after 1506 trials searching for 16 sites with resolution cut-off of 3.0 Å. The CFOM of the best solution was 64.9, CC_{all} was 41.4 and CC_{weak} was 23.5 (Supplementary Figure 11). The combined model building and refinement finished after 432 residues were built in 11 fragments, 89.6 % of them were assigned to the sequence (Supplementary Figure 12). The refinement R_{work} and R_{free} factors of the automatically built A_{2A} model were 30.4 % and 33.8 % respectively. The average peak height of the phased anomalous Fourier difference map for the first 17 highest peaks obtained with the fully refined model was 12.0.

The structure of A_{2A} from the minimal number of indexed images (50,000) was also determined to 2.65 Å resolution using the *CRANK2* pipeline. The heavy atom substructure was found by *SHELXD* after 18921 trials searching for 12 sites with resolution cut-off of 3.5 Å with additional parameters ESEL = 1.3 and MIND = {-3.5, 2.8} similarly to the substructure solution protocol for thaumatin 6.06 keV data set with minimal number of images. The CFOM of the best solution was 39.1, CC_{all} was 29.4 and CC_{weak} was 10.0 (Supplementary Figure 13). The combined model building and refinement finished after 403 residues were built in 12 fragments, 73.2 % of them were assigned to the sequence (Supplementary Figure 14). The refinement R_{work} and R_{free} factors of the automatically built A_{2A} model were 34.6 % and 43.2 % respectively. The average peak height of the phased anomalous Fourier difference map for the first 17 highest peaks obtained with the fully refined model was 9.48.

Table S1 Overall data quality indicators from CrystFEL of the thaumatin 6.06 keV data sets.

Number of images	Overall Rsplit [%]	Overall CC 1/2	Overall CC*	Overall CCano	SNR
271609	1.97	0.999	0.999	0.640	23.96
50000	4.81	0.998	0.999	0.296	11.81

Table S2 Data quality indicators from CrystFEL of the thaumatin 6.06 keV data set in resolution shells for all 271609 indexed images.

Resolution	Min	Max	Number	Redundancy	Rsplit [%]	CC 1/2	CC*	CC ano	SNR

shell	resolution [Å]	resolution [Å]	of reflections						
1	24.39	4.80	2458	3081	1.87	0.999	1.000	0.641	55.36
2	4.80	3.81	2462	1892	1.27	1.000	1.000	0.706	71.80
3	3.81	3.33	2438	1717	1.57	0.999	1.000	0.510	57.93
4	3.33	3.03	2423	1507	1.94	0.999	1.000	0.491	44.13
5	3.03	2.81	2444	1148	2.88	0.999	1.000	0.432	30.94
6	2.81	2.65	2437	1075	3.65	0.998	1.000	0.362	23.90
7	2.65	2.51	2437	1042	4.92	0.997	0.999	0.300	18.81
8	2.51	2.40	2446	1008	6.13	0.993	0.998	0.196	15.69
9	2.40	2.31	2456	928	7.61	0.992	0.998	0.212	12.16
10	2.31	2.23	2432	850	9.97	0.988	0.997	0.180	9.48
11	2.23	2.16	2416	857	13.14	0.975	0.994	0.121	7.42
12	2.16	2.10	2446	823	17.79	0.972	0.993	0.197	5.23
13	2.10	2.05	2445	636	29.48	0.958	0.989	0.197	3.06
14	2.05	2.00	2444	399	55.10	0.688	0.903	0.096	1.75
15	2.00	1.95	2384	280	134.86	0.732	0.919	0.279	0.65

Table S3 Data quality indicators from CrystFEL of the thaumatin 6.06 keV data set in resolution shells for 50000 indexed images.

Resolution shell	Min Resolution [Å]	Max resolution [Å]	Number of reflections	Redundancy	Rsplit [%]	CC 1/2	CC*	CC ano	SNR
1	24.39	4.92	2278	606	4.25	0.996	0.999	0.308	23.74
2	4.92	3.91	2267	364	2.86	0.998	1.000	0.396	31.90
3	3.91	3.42	2262	336	3.47	0.997	0.999	0.157	26.57
4	3.42	3.11	2270	301	4.09	0.996	0.999	0.209	21.42

5	3.11	2.88	2261	240	5.60	0.992	0.998	0.140	16.11
6	2.88	2.71	2270	197	7.59	0.990	0.998	0.169	12.18
7	2.71	2.58	2255	177	9.04	0.988	0.997	0.122	10.02
8	2.58	2.47	2243	150	11.08	0.980	0.995	-0.010	8.32
9	2.47	2.37	2265	126	13.18	0.973	0.993	0.085	7.22
10	2.37	2.29	2263	95	16.54	0.962	0.990	0.045	5.61
11	2.29	2.22	2260	77	20.91	0.937	0.984	0.081	4.51
12	2.22	2.15	2257	64	26.03	0.914	0.977	-0.008	3.61
13	2.15	2.10	2261	48	35.24	0.850	0.959	0.058	2.75
14	2.10	2.05	2261	28	52.26	0.774	0.934	0.144	1.81
15	2.05	2.00	2155	13	95.33	0.451	0.788	-0.009	1.17

Table S4 Overall data quality indicators from CrystFEL of the thaumatin 4.57 keV data sets.

Number of images	Overall Rsplit [%]	Overall CC 1/2	Overall CC*	Overall CCano	SNR
242578	1.72	0.999	0.999	0.877	34.97
20000	5.93	0.995	0.999	0.387	11.14

Table S5 Data quality indicators from CrystFEL of the thaumatin 4.57 keV data set in resolution shells for all 242578 indexed images.

Resolution shell	Min Resolution [Å]	Max Resolution [Å]	Number of reflections	Redundancy	Rsplit [%]	CC 1/2	CC*	CC ano	SNR
1	35.71	6.52	1050	3336	1.83	0.999	1.000	0.874	54.12
2	6.52	5.18	1042	2052	1.30	1.000	1.000	0.943	70.11
3	5.18	4.53	1022	1945	1.16	1.000	1.000	0.878	75.69
4	4.53	4.12	1045	1769	1.30	1.000	1.000	0.835	69.99
5	4.12	3.82	1019	1378	1.57	0.999	1.000	0.800	55.85
6	3.82	3.60	1041	1284	1.71	0.999	1.000	0.776	49.92
7	3.60	3.42	1020	1229	2.20	0.999	1.000	0.660	40.08
8	3.42	3.27	1030	1174	2.62	0.999	1.000	0.642	33.00
9	3.27	3.14	1012	1096	3.57	0.998	1.000	0.629	24.89
10	3.14	3.03	1047	969	4.90	0.996	0.999	0.406	18.34
11	3.03	2.94	1029	967	6.69	0.994	0.999	0.210	12.86
12	2.94	2.85	1020	945	9.29	0.992	0.998	0.358	9.04
13	2.85	2.78	1047	785	13.66	0.982	0.995	0.262	6.32
14	2.78	2.71	998	100	27.25	0.977	0.994	0.184	3.02
15	2.71	2.65	1066	348	75.81	0.864	0.963	0.207	0.99

Table S6 Data quality indicators from CrystFEL of the thaumatin 4.57 keV data set in resolution shells for 20000 indexed images.

Resolution Shell	Min resolution [Å]	Max resolution [Å]	Number of reflections	Redundancy	Rsplit [%]	CC 1/2	CC*	CC ano	SNR
1	35.71	6.52	1050	273	6.65	0.988	0.997	0.366	15.89
2	6.52	5.18	1042	165	4.42	0.996	0.999	0.611	20.60
3	5.18	4.53	1022	157	3.96	0.996	0.999	0.418	23.76
4	4.53	4.12	1045	147	4.06	0.997	0.999	0.289	22.41
5	4.12	3.82	1019	359	4.76	0.995	0.999	0.375	3.96
6	3.82	3.60	1041	101	6.00	0.993	0.998	0.133	15.52
7	3.60	3.42	1020	99	6.58	0.992	0.998	0.262	13.01
8	3.42	3.27	1030	95	8.40	0.988	0.997	0.211	10.70
9	3.27	3.14	1012	90	10.82	0.982	0.995	0.177	8.33
10	3.14	3.03	1047	80	14.90	0.970	0.992	0.186	6.14
11	3.03	2.94	1029	76	20.77	0.940	0.984	0.163	4.55
12	2.94	2.85	1020	76	27.70	0.894	0.972	0.181	3.22
13	2.85	2.78	1047	72	40.17	0.875	0.966	0.101	2.25
14	2.78	2.71	998	57	62.32	0.747	0.925	0.084	1.50
15	2.71	2.65	1066	37	111.57	0.856	0.961	0.049	0.79

Table S7 S_{ano} values obtained from the thatumaitn 4.57 keV data set processed by different versions of CrystFEL and refinement protocols. A clear increase in S_{ano} is visible between CrystFEL versions 0.7.0 and 0.8.0 only when the partiality refinement in *partialator* was used. The S_{ano} is the same when using or not using scaling with partiality refinement in both versions (S_{ano} = 8.96 and S_{ano} = 9.61). The Sano improved slightly when adding post-refinement to partiality refinement in the 0.8.0 version but decreased significantly in the 0.7.0 version. This indicates that scaling did not have any influence on the data quality and post-refinement improved it only slightly.

S_{ano} CrystFEL 0.7.0	S_{ano} CrystFEL 0.8.0	Partiality refinement	Post-refinement	Scaling	*partialator* parameters
7.88	9.88	+	+	+	--model=xsphere --iterations=1
8.96	9.61	+	-	+	--model=xsphere --iterations=1 –no-pr
8.96	9.61	+	-	-	--model=xsphere --iterations=0
8.80	8.77	-	-	+	--model=unity --iterations=1
8.80	8.77	-	-	-	--model=unity --iterations=0

Table S8 Overall data quality indicators from CrystFEL of the A_{2A} 4.57 keV data sets.

Number of images	Overall Rsplit [%]	Overall CC 1/2	Overall CC*	Overall CCano	Overall SNR
199123	2.26	0.999	0.999	0.568	23.29
50000	4.61	0.997	0.999	0.318	12.70

Table S9 Data quality indicators from CrystFEL of the A_{2A} 4.57 keV data set in resolution shells for all 199123 indexed images.

Resolution shell	Min resolution [Å]	Max resolution [Å]	Number of reflections	Redundancy	Rsplit [%]	CC 1/2	CC*	CC ano	SNR
1	35.71	6.52	1973	2305	2.20	0.999	1.000	0.535	48.33
2	6.52	5.18	1953	1317	1.91	0.999	1.000	0.800	47.51
3	5.18	4.53	1962	1228	1.61	0.999	1.000	0.708	56.25
4	4.53	4.12	1971	1108	1.85	0.999	1.000	0.602	50.14
5	4.12	3.82	1966	872	2.31	0.999	1.000	0.531	37.65
6	3.82	3.60	1936	743	3.17	0.998	0.999	0.450	28.57
7	3.60	3.42	1968	741	3.67	0.998	1.000	0.430	22.85
8	3.42	3.27	1961	719	4.88	0.996	0.999	0.366	17.58
9	3.27	3.14	1957	701	7.16	0.993	0.998	0.282	12.56
10	3.14	3.03	1943	619	9.85	0.987	0.997	0.159	9.42
11	3.03	2.94	1931	591	12.95	0.980	0.995	0.152	6.94
12	2.94	2.85	1970	601	18.16	0.967	0.992	0.191	5.13
13	2.85	2.78	1978	577	31.05	0.928	0.981	0.102	3.21
14	2.78	2.71	1922	454	55.17	0.866	0.963	0.112	1.72
15	2.71	2.65	1960	293	135.73	0.707	0.910	0.086	0.70

Table S10 Data quality indicators from CrystFEL of the A_{2A} 4.57 keV data set in resolution shells for 50000 indexed images.

Resolution shell	Min Resolution [Å]	Max resolution [Å]	Number of reflections	Redundancy	Rsplit [%]	CC 1/2	CC*	CC ano	SNR
1	35.71	6.52	1973	595	4.46	0.996	0.999	0.300	23.90
2	6.52	5.18	1953	342	3.82	0.997	0.999	0.536	24.31
3	5.18	4.53	1962	319	3.26	0.997	0.999	0.419	29.37
4	4.53	4.12	1971	289	3.51	0.997	0.999	0.352	26.61
5	4.12	3.82	1966	228	4.35	0.996	0.999	0.386	20.27
6	3.82	3.60	1936	195	5.75	0.993	0.998	0.195	15.72
7	3.60	3.42	1968	191	6.88	0.992	0.998	0.215	12.76
8	3.42	3.27	1961	178	8.73	0.988	0.997	0.237	10.05
9	3.27	3.14	1957	161	11.84	0.980	0.995	0.156	7.52
10	3.14	3.03	1943	127	15.18	0.966	0.991	0.081	5.93
11	3.03	2.94	1931	100	20.37	0.944	0.985	0.023	4.61
12	2.94	2.85	1970	76	25.13	0.922	0.979	0.085	3.72
13	2.85	2.78	1978	52	36.26	0.847	0.958	0.096	2.63
14	2.78	2.71	1922	29	55.52	0.690	0.904	0.079	1.76
15	2.71	2.65	1890	13	109.68	0.453	0.790	0.073	0.97

(a)

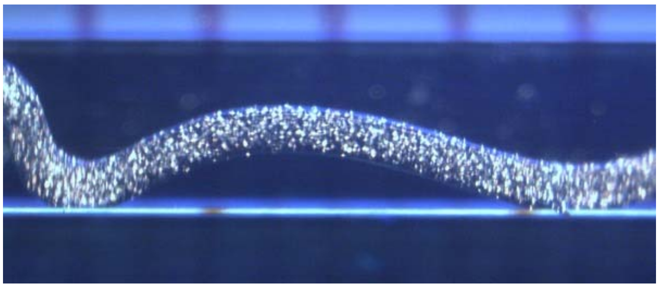

(b)

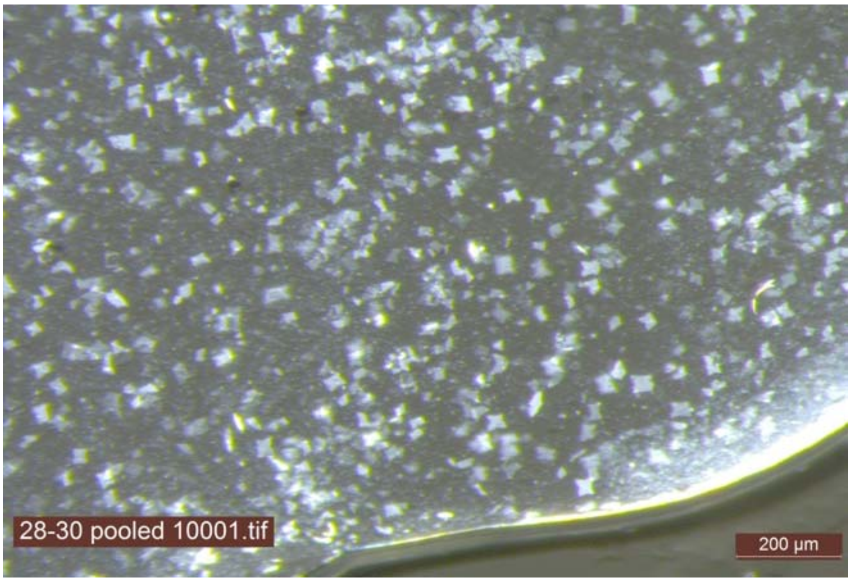

Figure S1 A_{2A} crystals grown in Hamilton syringe (a) and an image of the A_{2A} crystals grown in LCP (b).

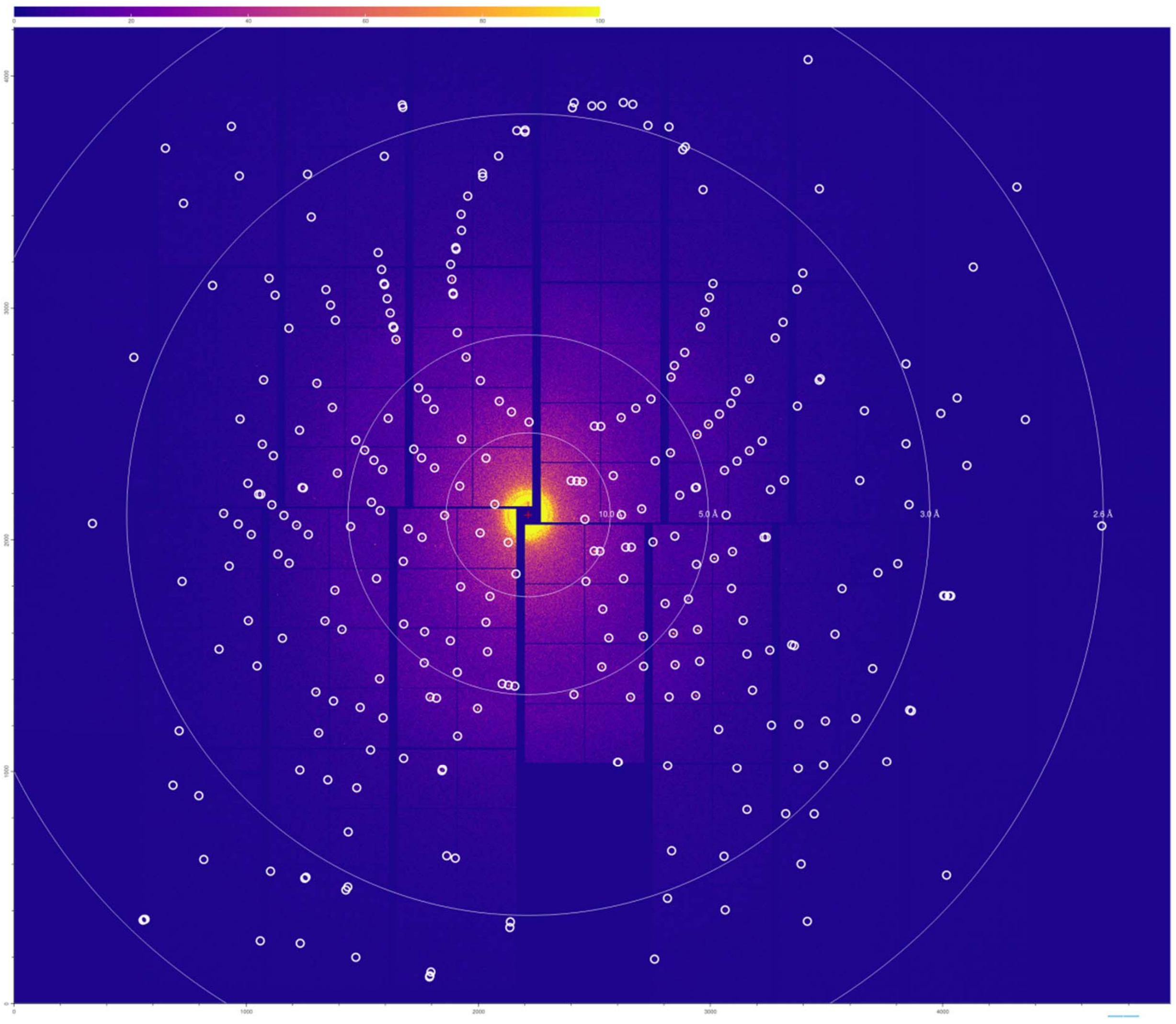

Figure S2 Diffraction image from an A_{2A} crystal recorded by the JUNGFRAU 16M detector. Small white circles indicate the positions of Bragg spot candidates found by the custom online hit detection tool.

Figure S3 Plots of the observed and calculated partialities for one of the thaumaitn crystals from 4.57 keV data set before and after partiality refinement (a, e). Contour plots of the R-wave (b, f), ang1-ang2 (e, g), ang1-wave (d, h) parameters before and after partiality and post-refinement.

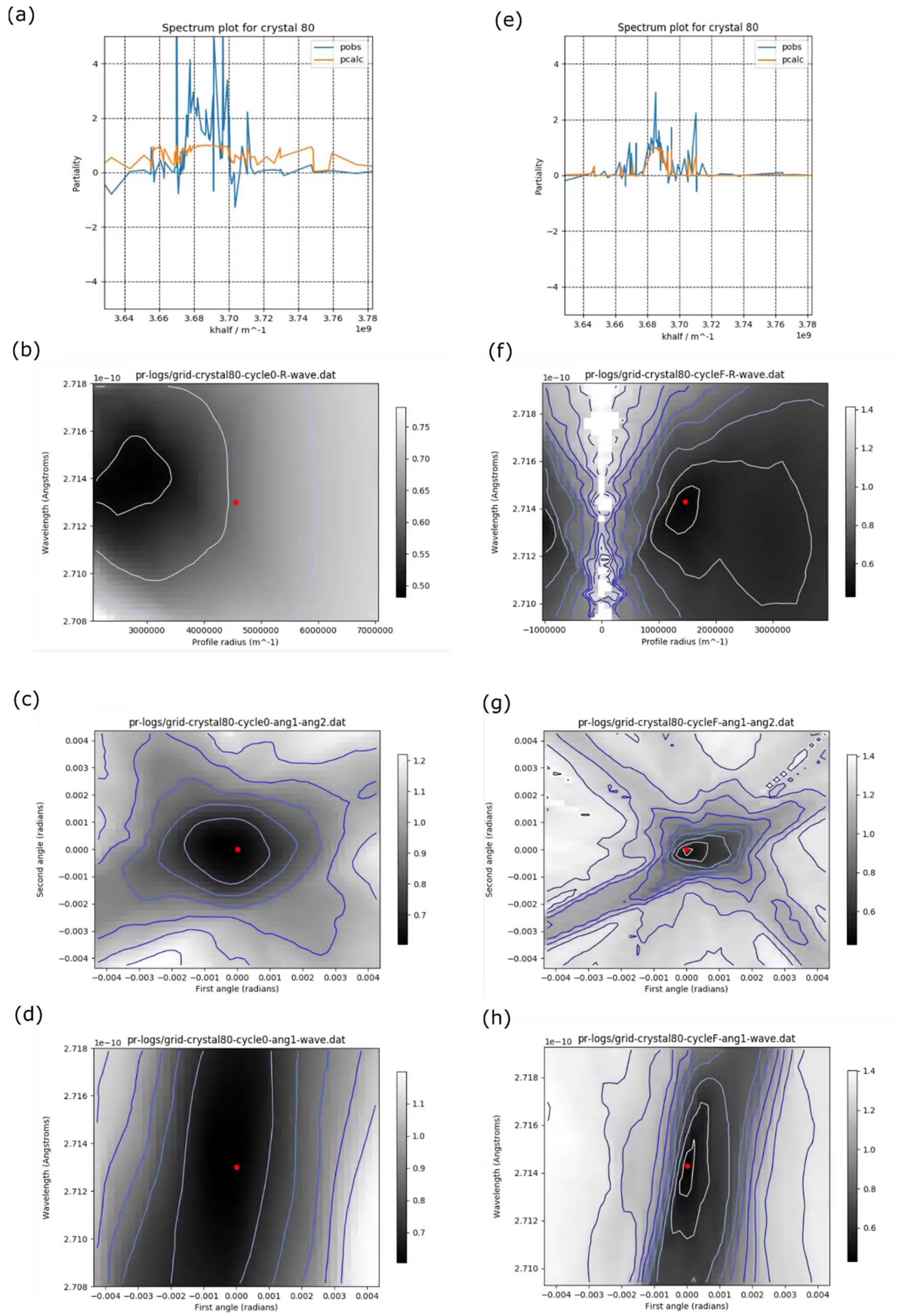

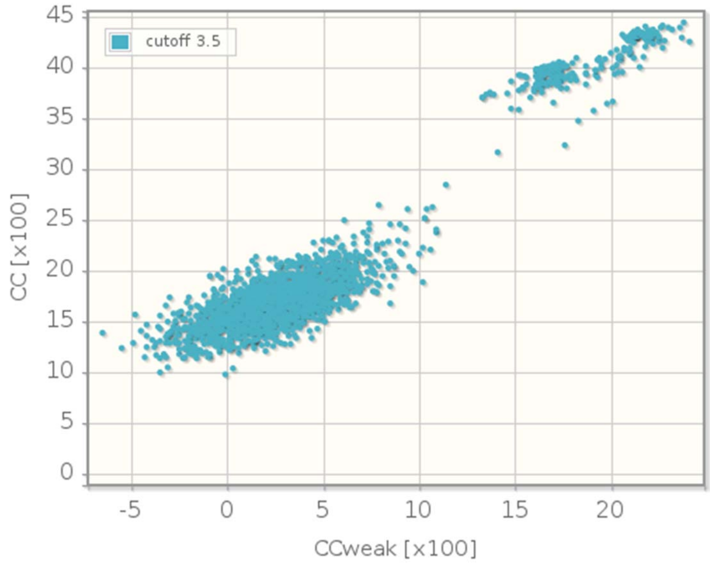

Figure S4 Histogram of CCall vs. CCweak values from SHELXD for thaumatin 6.06 keV using all indexed images.

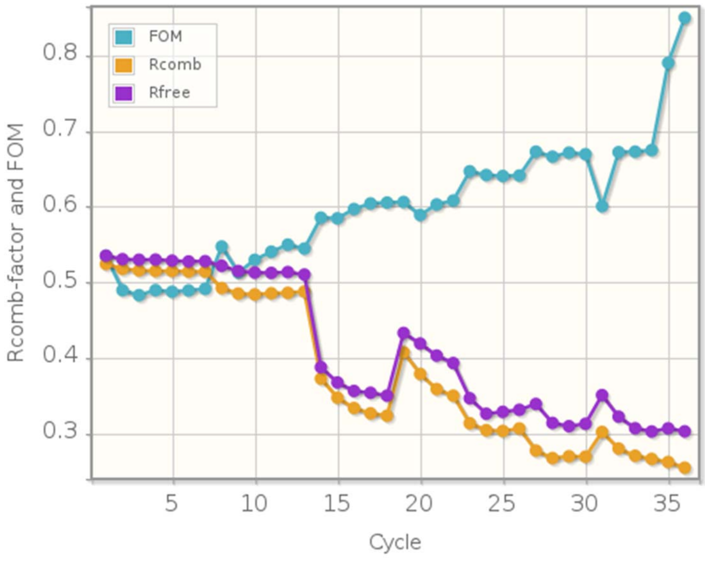

Figure S5 Progress of the combined model building and refinement of thaumatin 6.06 keV for all available indexed images.

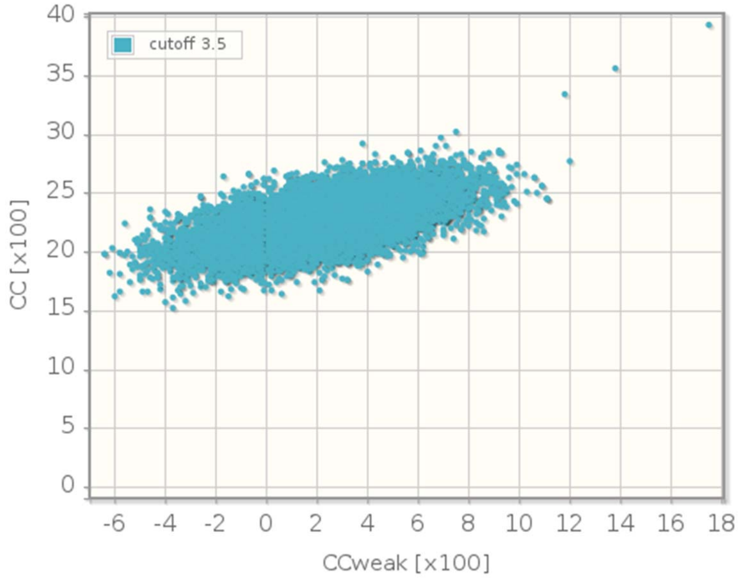

Figure S6 Histogram of CCall vs. CCweak values from SHELXD for thaumatin 6.06 keV using 50,000 indexed images.

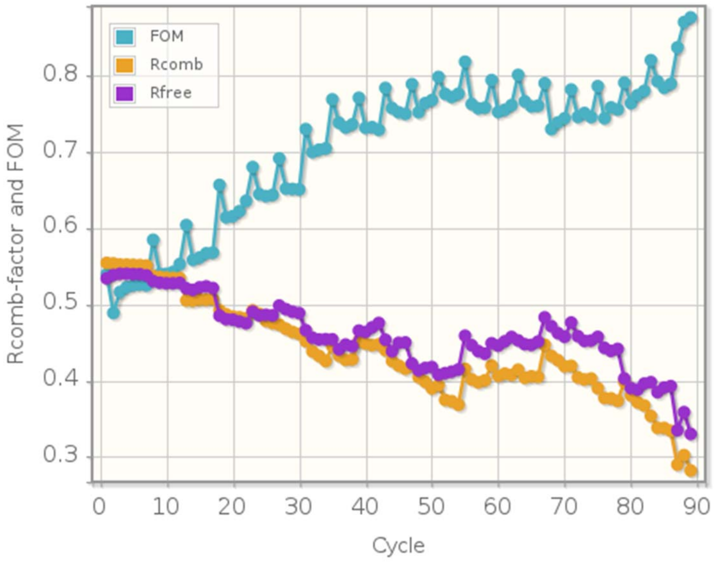

Figure S7 Progress of the combined model building and refinement of thaumatin 6.06 keV for 50,000 indexed images.

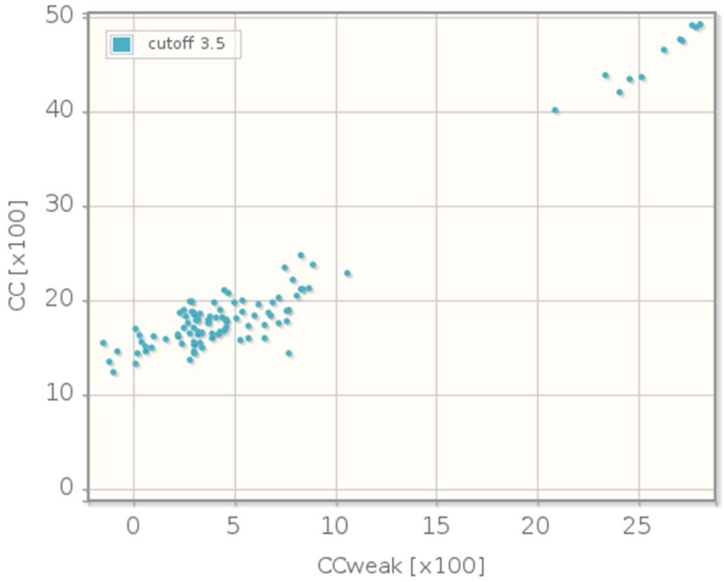

Figure S8 Histogram of CCall vs. CCweak values from SHELXD for thaumatin 4.57 keV using all available indexed images.

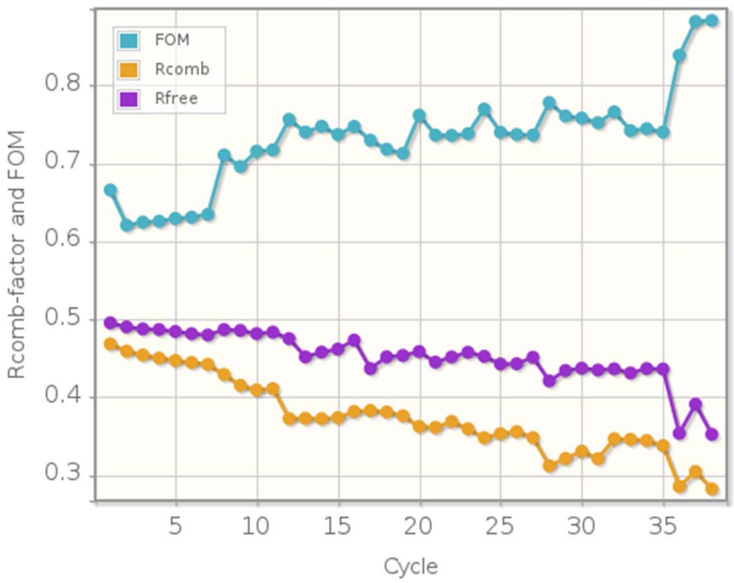

Figure S9 Progress of the combined model building and refinement of thaumatin 4.57 keV all available indexed images.

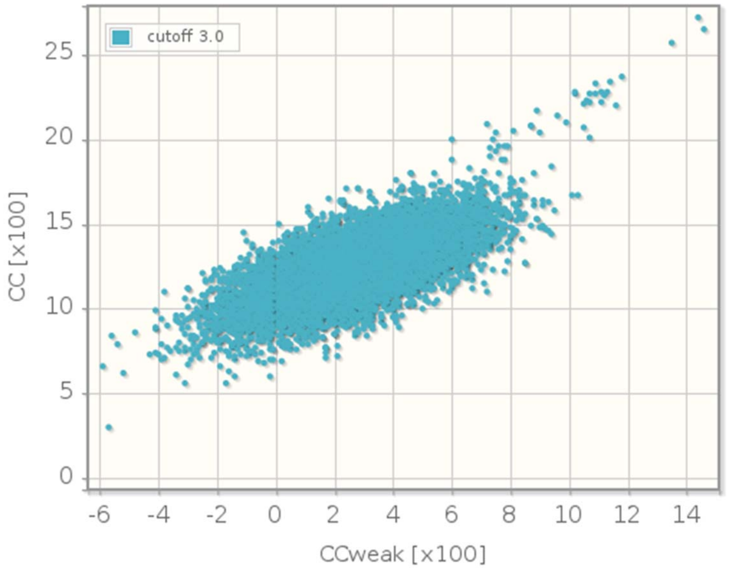

Figure S10 Histogram of CCall vs. CCweak values from SHELXD for thaumatin 4.57 keV 20,000 indexed images.

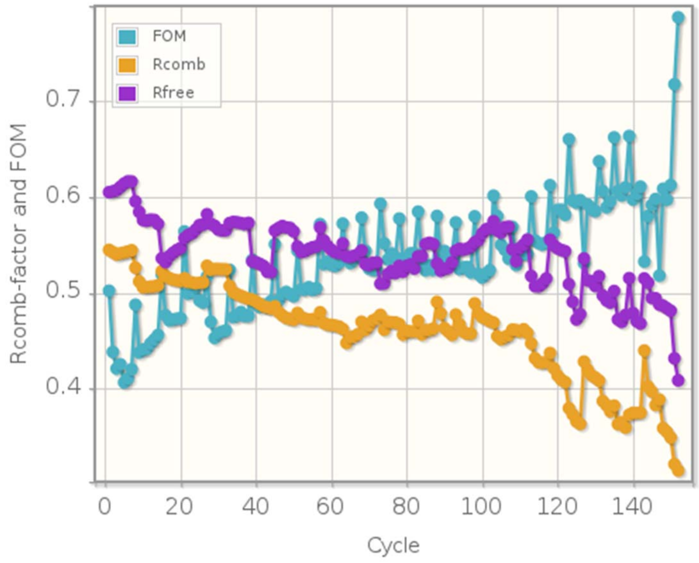

Figure S11 Progress of the combined model building and refinement of thaumatin 4.57 keV for 20,000 indexed images.

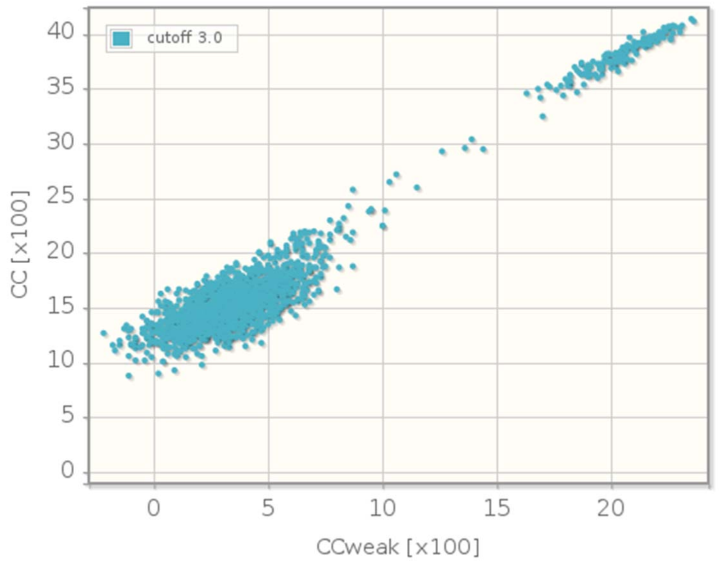

Figure S12 Histogram of CCall vs. CCweak values from *SHELXD* for A_{2A} using all available indexed images.

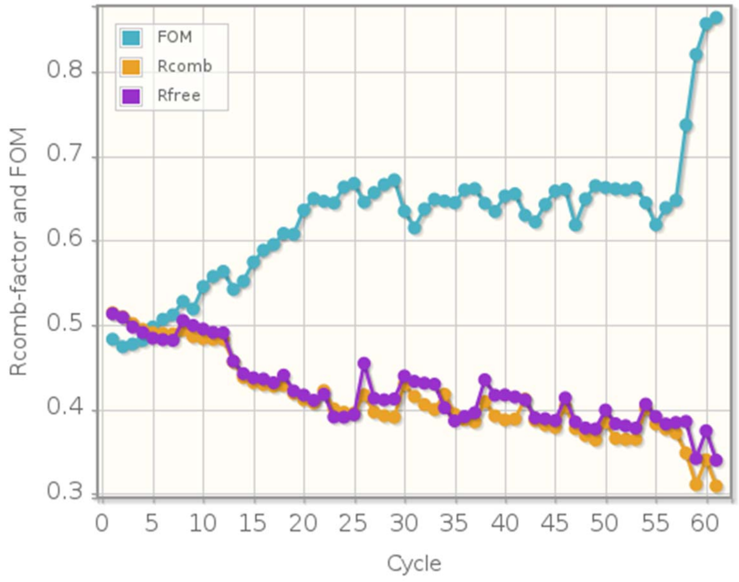

Figure S13 Progress of the combined model building and refinement of A_{2A} for all available indexed images.

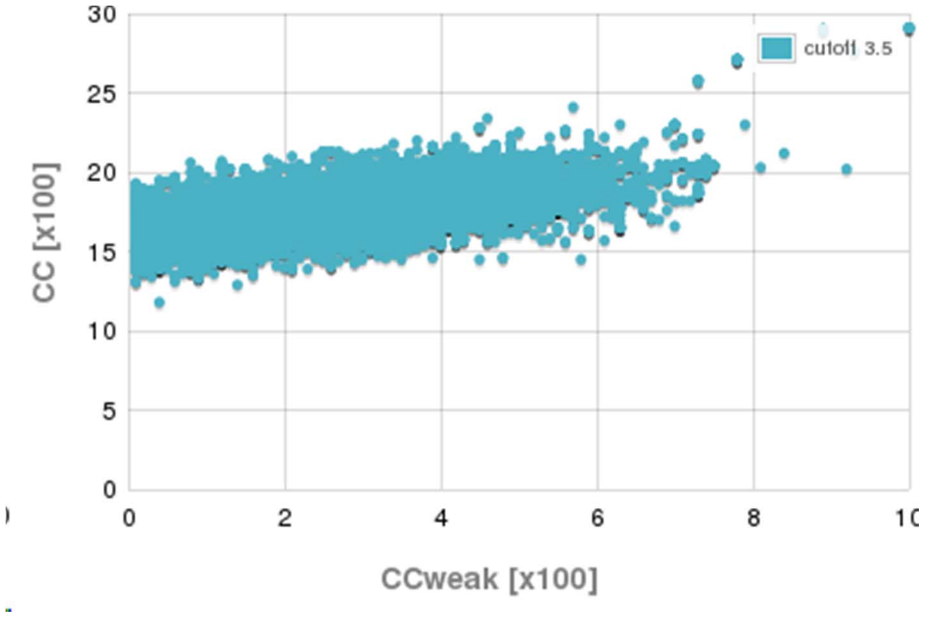

Figure S14 Histogram of CCall vs. CCweak values from SHELXD for A_{2A} using 50,000 indexed images.

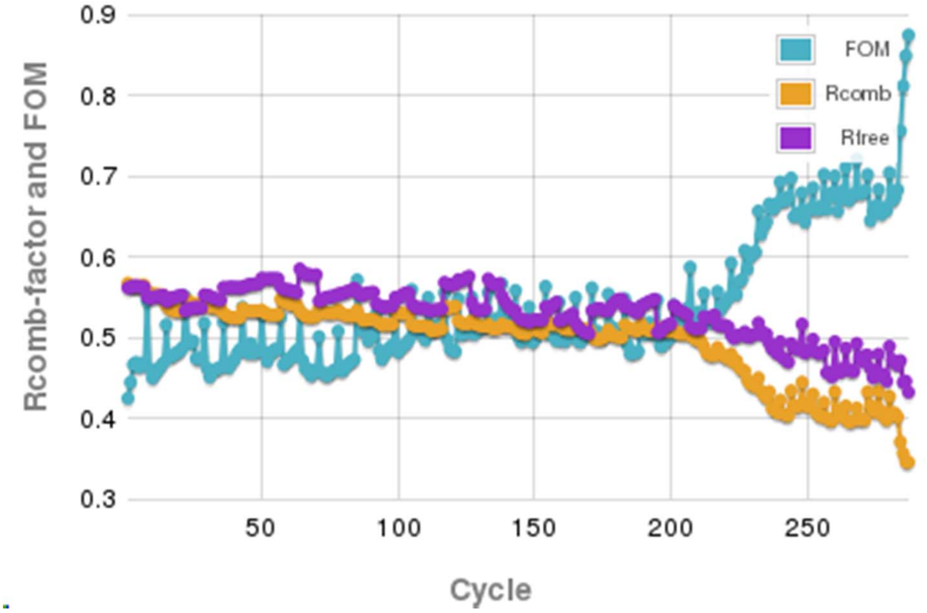

Figure S15 Progress of the combined model building and refinement of A2A for 50,000 indexed images.
